# Unklare Minderung des Allgemeinzustandes bei Patientin mit Lungenkarzinom

**DOI:** 10.1007/s00108-023-01652-5

**Published:** 2024-01-19

**Authors:** J. Brägelmann, S. Becker, D. Stenzel, A. Budahn

**Affiliations:** 1 Klinik für Endokrinologie, Diabetologie und Stoffwechsel, Universitätsmedizin Essen, Hufelandstraße 55, 45147 Essen, Deutschland; 2 Marienhaus Klinikum Mainz, Mainz, Deutschland

**Keywords:** Durvalumab, Immunbedingte unerwünschte Ereignisse (ibUE), Immun-Checkpoint-Inhibitoren-induzierter Typ-1-Diabetes (ICI-T1D), Elektronische Patientenakte (ePA), Informationsinfrastruktur, Durvalumab, Immune-Related Adverse Events (irAEs), Checkpoint inhibitor associated autoimmune diabetes mellitus (CIADM), Electronic patient file (epf), Information infrastructure

## Abstract

Eine 63-jährige Patientin mit Lungenkarzinom stellte sich erstmalig in unserer Notaufnahme mit plötzlicher Reduktion des Allgemeinzustands, Erbrechen und ausgeprägter Schwäche vor. Sie gab an, aufgrund des Lungenkarzinoms eine Chemotherapie zu erhalten, und verneinte weitere, relevante Vorerkrankungen. Unsere initiale Verdachtsdiagnose waren eine zytostatikainduzierte Nausea und Emesis. Die in der Notaufnahme durchgeführte Diagnostik erbrachte entgegen dieser Verdachtsdiagnose die Befunde einer Ketoazidose auf dem Boden einer Erstmanifestation eines Diabetes mellitus mit hyperglykämischer Entgleisung sowie einer schweren, manifesten Hypothyreose. Nach Beschaffung der Vorbefunde wurde evident, dass die Patientin keine Chemotherapie, sondern eine Immuncheckpointtherapie mittels Durvalumab erhielt. Die beschriebenen Erstmanifestationen waren demzufolge als durvalumabassoziierte Immunreaktionen zu werten. Nach Einleitung einer diabetischen Rekompensationstherapie und Substitution mittels L‑Thyroxin konnte eine rasche Verbesserung des Allgemeinzustands erreicht werden. Wären für uns relevante Vorbefunde schon zu Behandlungsbeginn abrufbar gewesen, beispielsweise durch die elektronische Patientenakte, wären die korrekte Interpretation der Symptome und die korrekte Therapie früher möglich gewesen.

## Abstract (en)

A 63-year-old female patient with lung cancer presented to our emergency room for the first time with a sudden reduction in general condition, vomiting and severe weakness. She stated that she was receiving chemotherapy for the lung cancer and reported that she had no other relevant previous illnesses. Our initial suspected diagnosis was cytostatic-induced nausea and vomiting. Contrary to this suspected diagnosis, diagnostics carried out in the emergency room revealed the findings of ketoacidosis on the basis of an initial manifestation of diabetes mellitus with hyperglycemic decompensation as well as severe, manifest hypothyroidism. After obtaining the preliminary findings, it became evident that the patient was not receiving chemotherapy, but rather immune checkpoint therapy using durvalumab. The initial manifestations described were therefore to be viewed as immune reactions associated with durvalumab. After initiating diabetic recompensation therapy and substitution with L‑thyroxine, a rapid improvement in the patientʼs general condition was achieved.

## Anamnese

Eine 63-jährige Patientin stellte sich über den Rettungsdienst mit ausgeprägter Fatigue, Appetitlosigkeit, Übelkeit, Erbrechen, Gewichtsverlust und Polydipsie seit zwei Wochen in der Notaufnahme vor. Sie berichtete, dass die Symptome so ausgeprägt gewesen seien, dass sie das Bett kaum noch habe verlassen können und „selbst das Sprechen zu anstrengend“ gewesen sei. Zudem hätten Fieber und Nachtschweiß bestanden. Dyspnoe und pektanginöse Beschwerden verneinte sie.

Bis zu dem Symptombeginn zwei Wochen zuvor habe sie ohne größere gesundheitliche Einschränkungen ihren Alltag bestreiten können einschließlich handwerklicher und sportlicher Tätigkeiten (Walken, Tennis).

Auf die Frage nach Vorerkrankungen gab sie an, an einem Lungenkarzinom zu leiden, welches mittels Chemo- und Radiotherapie behandelt werde, da der Tumor als derzeit inoperabel eingestuft worden sei. Die Erstdiagnose sei sechs Monaten zuvor erfolgt. Weder der Name des Chemotherapeutikums noch die genaue Anzahl und Intensität der stattgehabten Bestrahlungen waren ihr erinnerlich. Auch zum Tumorstadium konnte die Patientin zum Aufnahmezeitpunkt keine genauen Angaben machen.

Neben der von ihrem Onkologen verabreichten Medikation habe keine weitere Dauermedikation bestanden, Allergien verneinte sie, sie habe alle empfohlenen Impfungen vorgenommen und sei in den letzten Wochen nicht verreist.

## Untersuchung

In der körperlichen Untersuchung präsentierte sich die Patienten in stark reduziertem Allgemeinzustand mit klinischen Zeichen einer Exsikkose und blassem Hautkolorit. Darüber hinaus waren keine pathologischen Befunde evident.

## Initiale Verdachtsdiagnose

Voranzustellen ist, dass die Patientin zum ersten Mal in unserer Klinik behandelt wurde und keine Arztbriefe mitbrachte, sodass keinerlei Vorbefunde vorlagen. Diese wurden umgehend aus der behandelnden onkologischen Praxis angefordert. Bei oben stehender Anamnese bestand daher als initiale Verdachtsdiagnose eine zytostatikaassoziierte Nausea und Emesis.

## Befunde und Vorbefunde

Sonographisch sahen wir passend zum klinischen Bild der Exsikkose eine kollaptische V. cava inferior, darüber hinaus ergaben sich keine wegweisenden Befunde. Das EKG war unauffällig.

Die Blutgasanalyse (BGA) zeigte eine grenzwertige metabolische Azidose (pH 7,33) bei einer Hyperglykämie (Glukose 629 mg/dl) und einen erniedrigten Natriumwert (126 mmol/l), siehe Tab. 1.

**Table 1:** 

BGA-Abnahmeart			Kapillär
„Flow rate“		l/min	0,0
pH BGA	7,3–7,45	–	*7,33 ↓*
pCO_2_	32–43	mmHg	33,8
pO_2_	71–104	mmHg	86,6
Bicarbonat	21–28	mmol/l	*18,0 ↓*
O_2_-Sättigung	95–99	%	96,9
Na	136–146	mmol/l	*126,0 ↓*
cK	3,6–4,8	mmol/l	4,7
cCA	1,15–1,35	mmol/l	1,25
CL	98–106	mmol/l	*96,0 ↓*
Glukose	70–105	mg/dl	*629,0 ↑*
Laktat	< 16	mg/dl	10,0
Aktuelle BE	−2–(+)3	mmol/l	*−7,0 ↓*
Standard BE	−3–(+)2	mmol/l	*−7,9 ↓*
tHB	12–16	g/dl	12,8
Oxy-Hb-Fraktion	94–96	%	95,0
Carboxy-Hb-Fraktion	0,0–0,8	%	*1,2 ↑*

Laborchemisch war eine manifeste Hypothyreose mit einem TSH-Wert von 79,8 µIE/ml mit einem fT3 von 0,52 pg/ml und einem fT4 von 0,32 ng/dl erkennbar. Außerdem bestand eine Hyperglykämie von 743 mg/dl bei einem HbA_1c_ von 9,1 % (Tab. 2). Zudem sahen wir leicht erhöhte Leberwerte und Nierenretentionsparameter (Tab. 3). Das Blutbild war unauffällig.

**Table 2:** 

TSH basal	0,35–5,50	µIE/ml	*79,87 ↑*
Freies T3	2,3–4,2	pg/ml	*0,52 ↓*
Freies T4	0,89–1,76	ng/dl	*0,32 ↓*
Glukose	74–106	mg/dl	*743,0 ↑
HbA_1c_	4,0–6,0	%	*9,1 ↑*
HbA_1c_ IFCC	20–42	mmol/mol	*76,0 ↑*

**Table 3:** 

CRP	< 5,0	mg/l	3,9
Kreatinkinase	34–145	U/l	*292 ↑*
Natrium	135–144	mmol/l	*123 ↓*
Kalium	3,5–5,5	mmol/l	4,5
Kalzium	2,18–2,60	mmol/l	2,48
Kreatinin	0,5–1,1	mg/dl	*1,20 ↑*
GFR (CKD-EPI)	–	ml/min	*48,23
Harnstoff	19–49	mg/dl	27
Harnsäure	3,1–7,8	mg/dl	3,5
Lipase	12–53	U/l	39
Bilirubin gesamt	0,3–1,2	mg/dl	1,2
LDH	120–246	U/l	*266 ↑*
Alkalische Phosphatase	45–129	U/l	88
Gamma-GT	< 38	U/l	*49 ↑*
ALT (GPT)	7–40	U/l	*58 ↑*
AST (GOT)	13–40	U/l	*47 ↑*

Die Urinuntersuchung zeigte deutlich erhöhte Ketone (Tab. 4).

**Table 4:** 

Mikroalbumin	< 20	mg/l	
Spezifisches Gewicht	1,001–1,035	–	≤ 1,005
pH im Urin	4,6‑8,0	–	5,5
Leuko	< 15	/µl	0
Nitrit	Negativ	–	Negativ
Urineiweiß	Spur	mg/dl	Negativ
Uringlukose	Negativ	mg/dl	≥ 1000
Keton	Negativ	–	*40 ↑*
Urobilinogen	< 2	mg/dl	0,2
Urinbilirubin	Negativ	mg/dl	Negativ
Ery	< 10	/µl	0

Zur weiteren Diagnostik wurde während des stationären Aufenthalts eine autoimmunserologische Untersuchung mit Bestimmung der diabetesassoziierten Antikörper sowie der Bestimmung von ACTH und Kortisol angeschlossen mit dem Ergebnis: Inselzellantikörper (ICA) 1:20+, Glutamat-Decarboxylase(GAD65)-Antikörper 1553+++ IE/ml, Tyrosin-Phosphatase(IA2)-Antikörper < 10 U/l, Insulinautoantikörper (IAA) < 0,04 U/ml, Zinktransporter-8-Antikörper < 10,0 und C‑Peptid < 0,5 mg/ml. Für ACTH und Kortisol zeigten sich Normwerte.

Nach Erhalt des Faxes mit den Vorbefunden aus der behandelnden onkologischen Praxis erhielten wir nicht nur Kenntnis über das exakte Krankheitsstadium, sondern auch darüber, dass die Patientin zum Zeitpunkt der Aufnahme keine Chemotherapie, sondern eine Immuncheckpointtherapie mittels Durvalumab erhielt.

## Diagnose

Durvalumabassoziierte Immunreaktionen

1. Diabetes mellitus, Typ „checkpoint inhibitor-associated autoimmune diabetes mellitus“ (CIADM) mit Entgleisung und Ketoazidose

2. Manifeste Hypothyreose

3. V. a. beginnende Hepatitis

## Therapie und Verlauf

Die Patientin wurde stationär auf unsere pneumologische Station aufgenommen. Bezüglich des CIADM wurde durch das konsiliarisch hinzugezogene Diabetesteam eine intensivierte konventionelle Insulintherapie (ICT) etabliert. Bei manifester Hypothyreose wurde eine Substitutionstherapie mit 125 µg L-Thyroxin p.o. 1‑0‑0 begonnen. Passager wurde intravenös Flüssigkeit substituiert.

Nach interdisziplinärer Falldiskussion wurde eine Fortführung der Konsolidierungstherapie unter engmaschigen Kontrollen empfohlen.

Unter der oben stehenden Therapie konnte rasch eine Rekompensation der endokrinen Entgleisungen erreicht werden. Hiernach wünschte die Patientin eine frühzeitige Entlassung ins häusliche Umfeld. Der Fortführung der Konsolidierungstherapie stand sie ablehnend gegenüber, da sie der Verlauf sehr verunsichert habe.

## Diskussion

Checkpointinhibitoren modifizieren die immunologische Eigentoleranz und werden zur medikamentösen Tumortherapie eingesetzt. Seit der Markteinführung haben sie die Überlebensdauer von Patientinnen und Patienten mit verschiedenen Tumorleiden erheblich verlängert und gelten als einer der wichtigsten Fortschritte in der Geschichte der Tumortherapie [8, 10].

Durvalumab, Handelsname Imfinzi, ist ein Immuncheckpointinhibitor, der als Antikörper spezifisch an den „programmed cell death ligand 1“ (PD-L1) bindet und diesen hemmt. Er wurde von AstraZeneca entwickelt und erhielt 2018 seine Zulassung in der Europäischen Union. Im ungehemmten, naiven Zustand interagiert PD-L1 mit dem „programmed cell death protein 1“ (PD-1) und dem Lymphozytenaktivierungsgen CD80, wodurch T‑Zellen gehemmt werden. Als Folge können sich Tumorzellen gegen das körpereigene Immunsystem schützen, was als Immunevasion bezeichnet wird. Durch den Einsatz von Durvalumab kann die Bindung von PD-L1/PD‑1 und PD-L1/CD80 verhindert werden, sodass die Bildung und Aktivität von T‑Zellen gegenüber Tumorzellen, also die körpereigene, Antitumorantwort, verstärkt wird [7, 8].

Die therapeutische Modifikation von Immunzellen anstatt von Tumorzellen selbst hat nicht nur eine Veränderung der pharmakologischen Wirkmechanismen und des Behandlungserfolgs nach sich gezogen, sondern auch eine Veränderung des Nebenwirkungsprofils. Das körpereigene Immunsystem kann in ein Ungleichgewicht geraten und iatrogene, autoimmune Erkrankungen können entstehen, welche als immunbedingte, unerwünschte Ereignisse („immun-related adverse events“ [IRAE]) bezeichnet werden [6].

Für PD-L1-Antikörper gelten autoimmune Schilddrüsenerkrankungen als häufige IRAE (Auftreten in 5,9 % der Therapien), wohingegen ein Typ-1-Diabetes (0–1 %) und eine Hypophysitis (< 2 %) selten sind [3, 4].

Das Lungenkarzinom der Patientin in unserer Kasuistik zeigte ein gutes Ansprechen auf Durvalumab, jedoch traten erhebliche autoimmune Nebenwirkungen auf, welche nach Substitution der entsprechenden peripher wirksamen Hormone erfolgreich behandelt werden konnten.

Eine Fortführung der Konsolidierungstherapie wurde der Patientin nach ausgiebiger Kosten-Nutzen-Abwägung empfohlen, von ihr jedoch abgelehnt. Die oben genannten Nebenwirkungen mit der entsprechenden, ausgeprägten Klinik und die Unordnung über die initiale Einordnung der Genese ihrer Beschwerden hätten bei ihr eine große Verunsicherung ausgelöst. Der Abbruch der Konsolidierungstherapie des inoperablen Plattenepithelkarzinoms durch die Patientin, trotz guten onkologischen Ansprechens, schien einen Wendepunkt ihrer onkologischen Prognose einzuläuten.

Die aufgetretenen IRAE unter Durvalumab zählen zu zwar seltenen, aber bekannten und pharmalogisch schlüssigen Nebenwirkungen und hätten als solche von den Behandelnden nicht vermieden werden können.

Was jedoch hätte vermieden werden können, war das große Informationsdefizit zu Behandlungsbeginn, welches für die ärztlichen und pflegerischen Teams der Notaufnahme und der behandelnden onkologischen Praxis Mehrarbeit durch telefonischen Informationsaustauch und das Faxen von Inhalten bedeutete und bei der Patientin die aufgrund des Tumorleidens ohnehin schon erhebliche Verunsicherung verstärkte und schlussendlich zum Behandlungsabbruch beitrug.

Hier hätte die Möglichkeit zum bedarfsgerechten Abrufen von zentral und elektronisch gespeicherten Gesundheitsdaten für die Behandelnden und die Patientin die Einordnung der Akutsituation in den medizinischen Gesamtkontext erheblich erleichtert und beschleunigt.

Seit dem 01.01.2021 steht mir der Einführung der elektronischen Patientenakte (ePA) in Deutschland grundsätzlich ein solches Werkzeug zur Verfügung. Für alle gesetzlich Versicherten besteht die Möglichkeit, per Opt-in-Regelung eine ePA zu beantragen. Nach Angaben der Gesellschaft für Telematikanwendungen der Gesundheitskarte mbH (gematik) verfügen jedoch derzeit (Stand 19.11.2023) lediglich 866.566 gesetzlich Versicherte über eine ePA, was einem Anteil von lediglich einem bis zwei Prozent aller gesetzlich Versicherten in Deutschland entspricht [5]. Durch diesen verschwindend kleinen prozentualen Nutzungsanteil ist die ePA somit derzeit in der klinischen Praxis kaum von Bedeutung.

Was zeigt der Blick ins europäische Ausland? Bereits 2018 untersuchte eine von der Bertelsmann Stiftung in Auftrag gegebene Studie zu internationalen Digitalisierungsstrategien die Bedingungen für Sekundärdatennutzungen in 17 verschiedenen Ländern. Als Grundlage dienten hierbei 34 Indikatoren zu Digitalisierungsstrategie, Verfügbarkeit und digitaler Ausreifung. Deutschland belegte hierbei den vorletzten Platz, zu den Spitzenreitern gehörte Dänemark [9].

In Dänemark wurde erstmalig im Jahre 1999 eine elektronische Gesundheitsstrategie beschlossen, heute ist der zentrale Pfeiler der medizinischen Digitalisierung die nationale Gesundheitsplattform sundhed.dk, von der aus sowohl das medizinische Personal als auch die Patientinnen und Patienten selbst Daten abrufen können, zu denen sie eine Berechtigung haben. Heute werden elektronische Rezepte in Dänemark zu 100 % angeboten und zu 99 % genutzt. Die Nutzung der ePA durch die Patientinnen und Patienten selbst ist regional unterschiedlich stark ausgeprägt und beträgt 80 % in Süddänemark und nur 30 % in Mitteldänemark, was auf soziodemografische Unterschiede (insbesondere das Alter) zurückgeführt wird. Überweisungen werden zu 97 % elektronisch ausgestellt und medizinische Akten werden zu 98 % zwischen Ärztinnen und Ärzten elektronisch ausgetauscht. Das Vertrauen der dänischen Bevölkerung in medizinische Einrichtungen und die elektronische Datenspeicherung sei hoch [1, 9].

Wie könnte sich nun auch in Deutschland die Nutzung der bereits 2021 implementierten ePA vereinfachen und verbreiten lassen? Was könnte man beispielsweise vom Digitalisierungsspitzenreiter Dänemark lernen?

In ihrer aktuellen Stellungnahme zum Stand der ePA befürwortete die AG Digitale Versorgungsforschung der DGIM die Umstellung von der derzeitigen Opt-in-Regelung zu einem Widerspruchsverfahren (Opt-out), sodass zukünftig jede Patientin und jeder Patient eine ePA erhalten würde und aktiv widersprechen müsste, wenn dies nicht gewünscht wäre [2]. Zudem wäre eine breit angelegte, öffentliche Aufklärungskampagne sinnvoll, um Chancen und Nutzen der ePA in die Bevölkerung zu tragen.

Möglicherweise könnten so zukünftig wichtige Informationen wie in unserem Fall einfach digital abgerufen werden, wodurch eine deutliche Verschlankung des administrativen Aufwands und eine Steigerung der Patientensicherheit erreicht werden würden.

## Fazit für die Praxis

- Checkpointinhibitoren können autoimmuntoxische Nebenwirkungen haben – beachte hier auch seltenere Entitäten wie den Diabetes mellitus Typ 1!
- Wenn verschiedene Krankheitsbilder zeitgleich auftreten, sei wachsam – eine gemeinsame Ätiologie muss nicht auf den ersten Blick evident sein.
- Eine „Chemotherapie“ muss keine Chemotherapie sein – Patientinnen und Patienten kennen oft nicht alle Details zu ihrer Erkrankung und Therapie.
- Kläre über die elektronische Patientenakte auf – sie könnte beiden Seiten Sicherheit schenken und Arbeit ersparen.
- Eine Umstellung von der Opt-in- zu einer Opt-out-Regelung könnte einen Meilenstein der Digitalisierung im deutschen Gesundheitswesen bedeuten.
